# Supplementary material for: Re-evaluation of the psychometric properties of ATE following changes in euthanasia regulations in Spain
Source: PLoS One. 2025 Feb 13;20(2):e0319015. doi: 10.1371/journal.pone.0319015 (PMC11824957; doi:10.1371/journal.pone.0319015)
Supplement: S1 File — (PDF) [file pone.0319015.s001.pdf]

Escala ATE	Escala ATE-ES-R
1. If a patient in severe pain requests it, a doctor should remove life support and allow that patient to die.	1. Si un paciente con dolor severo lo solicita, el equipo asistencial debe eliminar el soporte vital y permitir que ese paciente muera.
2. It is okay for a doctor to administer enough medicine to end a patient’s life if the doctor does not believe that they will recover.	2. Está bien que el equipo asistencial administre suficientes medicamentos para terminar con la vida de un paciente si el equipo asistencial no cree que se recuperará.
3. If a patient in severe pain requests it, a doctor should prescribe that patient enough medicine to end their life.	3. Si un paciente con dolor severo lo solicita, el equipo asistencial debe recetarle suficiente medicamento al paciente para terminar con su vida.
4. It is okay for a doctor to remove life-support and let a patient die if the doctor does not believe the patient will recover.	4. Está bien que el equipo asistencial elimine el soporte vital y deje que el paciente muera si el equipo asistencial no cree que el paciente se recuperará.
5. It is okay for a doctor to administer enough medicine to a suffering patient to end that patient’s life if the doctor thinks that the patient’s pain is too severe.	5. Está bien que el equipo asistencial administre suficiente medicamento a un paciente que sufre para terminar con la vida de ese paciente si el equipo asistencial cree que el dolor del paciente es demasiado severo.
6. Even if a doctor does not think that a patient will NR recover, it would be wrong for the doctor to end the life of a patient.	6. Incluso si el equipo asistencial no cree que un paciente se recuperará, sería incorrecto que el equipo asistencial termine con la vida de un paciente.
7. It is okay for a doctor to remove a patient’s life-support and let them die if the doctor thinks that the patient’s pain is too severe.	7. Está bien que el equipo asistencial elimine el soporte vital de un paciente y lo deje morir si el equipo asistencial cree que el dolor del paciente es demasiado severo.
8. If a dying patient requests it, a doctor should prescribe enough medicine to end their life.	8. Si un paciente moribundo lo solicita, el equipo asistencial debe recetar suficientes medicamentos para terminar con su vida.
9. Even if a doctor knows that a patient is in severe, SP uncontrollable pain, it would be wrong for the doctor to end the life of that patient.	9. Incluso si el equipo asistencial sabe que un paciente tiene un dolor severo e incontrolable, sería incorrecto que el equipo asistencial termine la vida de ese paciente.
10. If a dying patient requests it, a doctor should remove their life support and allow them to die.	10. Si un paciente moribundo lo solicita, equipo asistencial debe quitarle el soporte vital y dejar que muera.

ATE-ES-R Scale
1. If a patient in severe pain requests it, the care team should remove life support and allow that patient to die.
2. It is okay for the care team to administer enough medicine to end a patient’s life if the care team does not believe that they will recover.

3. If a patient in severe pain requests it, the care team should prescribe that patient enough medicine to end their life.
4. It is okay for the care team to remove life-support and let a patient die if the care team does not believe the patient will recover.
5. It is okay for the care team to administer enough medicine to a suffering patient to end that patient’s life if the care team thinks that the patient’s pain is too severe.
6. Even if the care team does not think that a patient will NR recover, it would be wrong for the care team to end the life of a patient.
7. It is okay for the care team to remove a patient’s life-support and let them die if the care team thinks that the patient’s pain is too severe.
8. If a dying patient requests it, the care team should prescribe enough medicine to end their life.
9. Even if the care team knows that a patient is in severe and uncontrollable pain, it would be wrong for the care team to end the life of that patient.
10. If a dying patient requests it, the care team should remove their life support and allow them to die.
