# Supplementary material for: Re-evaluation of the psychometric properties of ATE following changes in euthanasia regulations in Spain
Source: PLoS One. 2025 Feb 13;20(2):e0319015. doi: 10.1371/journal.pone.0319015 (PMC11824957; doi:10.1371/journal.pone.0319015)
Supplement: Attachment — Submitted filename: Answers to Reviewers.docx [file pone.0319015.s002.docx]

**Response to Reviewer #1:**

We thank the reviewer for his/her vigorous appraisal of our work. In what follows we address his comments, point by point.

Abstract:

**1.- Reviewer’s comment**: “You make a very correct introduction to the topic, adequately stating the importance and relevance of the topic (Euthanasia). However, I believe that the relevance of this study is not adequately explained. It does not explain why it is necessary to re-evaluate the Attitude Toward Euthanasia scale (ATE), validated by Fernandez et al., of which some of you are also authors. It should be reflected what possible shortcomings the first version has that obliges to re-evaluate the instrument and what this manuscript contributes again (the values provided with respect to the psychometric properties evaluated in the first version are not bad). In fact, the objective that appears at the end of the introduction does not coincide with the one that appears at the beginning of the discussion”.

Authors’ response: Thank you for your insightful comments regarding the relevance of our study. We appreciate your acknowledgment of the importance of the topic of euthanasia. In response to your concerns, we have revised the introduction to more clearly articulate the necessity of reevaluating the Attitude Toward Euthanasia (ATE) scale originally validated by Fernandez et al.

The primary reasons for this reevaluation are as follows: First, the introduction of the Euthanasia Law in Spain has significantly altered the social and healthcare context in which attitudes toward euthanasia are formed. Given these changes, we thought it was very important to reassess the ATE scale to ensure that it remains relevant and accurately reflects current attitudes and perceptions.

Second, while the psychometric properties of the initial version of the ATE scale were acceptable, there were identified areas for improvement. For example, certain items may not adequately capture the nuances of contemporary attitudes shaped by recent legal and societal developments and some items were missed from the original version.

We have clarified these points in the revised text and ensured that the objectives stated at the beginning of the introduction align with those presented in the discussion.

The introduction of the Euthanasia Law in Spain has significantly altered the social and healthcare context in which attitudes toward euthanasia are formed. Given these changes, it is crucial to reassess the Attitude Toward Euthanasia (ATE) scale to ensure that it remains relevant and accurately reflects current attitudes and perceptions among healthcare professionals. While the psychometric properties of the initial version of the ATE scale were acceptable, areas for improvement were identified. For instance, certain items may not adequately capture the nuances of contemporary attitudes shaped by recent legal and societal developments. This study aims to address these gaps by validating and adapting the ATE scale for a diverse sample of healthcare professionals in SpainTherefore, the aim of this study has been to revise the first translation/validation of the Attitude Toward Euthanasia scale (ATE) by Fernandez et al. (29) using a larger and more heterogeneous sample of healthcare professionals. This approach will allow for a comprehensive understanding of attitudes across different roles within the healthcare sector. The study aims to identify the different dimensions of the questionnaire and to partially modify certain items to better align with the current cultural context in Spain, particularly in light of the regulation of euthanasia in 2021.Ultimately, the goal is to develop an instrument that will be assessed for its reliability and validity after the study is completed, reflecting the evolving attitudes of healthcare professionals toward euthanasia amidst recent legal changes

**2.- Reviewer’s comment**:” In the introduction, in the sentence: ‘The number of patients who request or receive MAID is gradually increasing in Spain, since the law was develop a few years ago,’ the meaning of the acronym MAID should be explained and serious references should be made to the law alluded to”.

Authors’ response: We appreciate the reviewer’s comments. We have made the suggested revisions to the text. In the introduction, we have clearly defined the meaning of the acronym MAID (Medical Assistance in Dying) to avoid any ambiguity. Additionally, we have included specific and up-to-date references to Organic Law 3/2021 regulating euthanasia in Spain, citing relevant literature to support our statement about the gradual increase in patients requesting or receiving MAID since the implementation of this law.

“There is an ongoing debate on the decriminalization of euthanasia in Spain, which tends to resurface following high-profile media cases. Medical Assistance in Dying (MAID) encompasses both the euthanasia process and assisted suicide, serving as the focal point in the current bioethical debate on end-of-life ethical dilemmas. Since the approval of Spain's Organic Law 3/2021 regulating euthanasia, the number of patients requesting or receiving MAID has been steadily increasing.(20–29). Although this law was developed a few years ago, its implications continue to shape discussions in both medical and ethical fields (30).”

**3- Reviewer’s comment**: “Study population. The study population should be better defined, as the term health worker is very broad, e.g. physiotherapists or psychologists participated? The total percentages of the professions to which they belonged are not provided in the results. This is important, because there may be differences between professions”.

Authors’ response: We appreciate the reviewer’s valuable feedback. We have clarified the study population by providing the specific percentages of the different professions included in our sample. To address the concern regarding variability among professions, we have refined our analysis by dividing the sample into two groups for conducting the EFA and CFA. We have also excluded undergraduate students from the analysis to ensure that all participants are active professionals, representing a wide range of occupations to enrich the study's results. See table 1

**4- Reviewer’s comment**: “Should justify with references the estimated sample size, which I agree is sufficient for a CFA, but if it is 10 subjects per item it does not correspond to the estimated size. The sample size for factor analysis is a highly controversial issue that I will not develop here (the concept of 10 subjects per item is nowadays very outdated, as the sample size will depend on the number of items, number of response ranges and other elements)”.

Authors’ response:

Thank you for your insightful comment regarding the sample size estimation for the confirmatory factor analysis (CFA). We acknowledge that the rule of thumb of 10 subjects per item has been widely debated in the literature, and indeed, it is considered outdated by many scholars in psychometrics and factor analysis. Recent studies suggest that sample size requirements should be guided by a combination of factors, including the number of items, the number of response categories, the expected factor loadings, and the complexity of the model (MacCallum et al., 1999; Kline, 2015; Wolf et al., 2013).

In light of these recommendations, we carefully evaluated the sample size in relation to these elements to ensure that it is sufficient to provide reliable and robust CFA results. Our approach aligns with current best practices in the field, which emphasize the importance of a tailored evaluation of sample adequacy rather than relying on rigid rules like the 10-to-1 ratio."

1. Kline, R. B. (2015). *Principles and Practice of Structural Equation Modeling* (4th ed.). The Guilford Press.
2. Wolf, E. J., Harrington, K. M., Clark, S. L., & Miller, M. W. (2013). Sample size requirements for structural equation models: An evaluation of power, bias, and solution propriety. *Educational and Psychological Measurement, 73*(6), 913-934.
3. Ferrando, P.J.; Lorenzo-Seva, U.; Hernández-Dorado, A.; Muñiz, J. Decalogue for the Factor Analysis of Test Items. Psicothema 2022, 34, 7–17.

**5- Reviewer’s comment**: “Information should be provided on the control mechanisms implemented to ensure the internal validity of the results, as this is an online survey. To which e-mail address was the invitation sent to potential participants? Also in the case of recruits, the response rate should be indicated”.

Authors’ response: Thank you for highlighting the importance of internal validity in online surveys. To ensure the reliability and validity of our results, we implemented several control mechanisms throughout the data collection process.

For the recruitment of the sample, once authorization was obtained from the various management bodies of the Balearic Health Service, collaboration was requested through the respective research committees to disseminate the questionnaire via a global corporate mailing to all professionals. Additionally, the questionnaire was made accessible on the intranet through a direct link.

Regarding the response rate, we have now included a detailed breakdown in the manuscript, specifying the number of invitations sent, the number of responses received, and the final response rate after applying our inclusion criteria. According to the census of healthcare professionals in the Balearic Community (https://www.ibsalut.es//es/profesionales/recursos_humanos/plantillas), the number of healthcare professionals serving in the Balearic Islands totals 5,400, which results in a response rate of 16.56%.

***Material and methods***

*Sample*

*All health professionals of the Balearic Health Service and specialist professionals in training in the Balearic Islands were invited to participate in the study. Convenience non-probabilistic sampling was used. The study did not consider probabilistic sampling as its primary objective was to achieve a large sample size.For the recruitment of the sample, once authorized by the different managements, through the respective research commissions, collaboration was requested for the dissemination of the questionnaire by global mailing to all the professionals of the different health services. The response rate for the study was 16.56%*

**6- Reviewer’s comment**: “One aspect that is not sufficiently explained is whether a new translation of the original English instrument has been carried out or whether it has been based on the version by Fernandez et al. It is also not clear how many translators were involved in each phase of translation and back-translation”.

Authors’ response: Thank you for your insightful comment. We would like to clarify that our study was based on the original English version of the Attitude Toward Euthanasia (ATE). Given the recent introduction of the Euthanasia Law in Spain, it is very important to revaluate and adapt certain concepts in the questionnaire to ensure their relevance in the current context. We conducted a new translation to enhance cultural and contextual applicability. We have now explained this in text with more details.

*Design*

*Based on the ATE scale (39,40), we conducted a new translation to enhance cultural and contextual applicability. The translation process involved two bilingual translators whose native language is Spanish. After the initial translation, a back-translation was performed by a professional translator whose native language is English. Subsequently, the research team reviewed the translated version and a second team, formed by members of the Health Care Ethics Committee of the Ibiza and Formentera Health Area, revised it to obtain a semantic and technical equivalence in appropriate bioethical language. In agreement with the Health Care Ethics Committee, it was agreed to change the expression “the physician” in all the items to “the health care team”, which better reflects how health care is carried out in our context and reflects the involvement of all the health care professionals in the process of death. Furthermore, although the text of Law 3/2021 regulating euthanasia in Spain (30) refers to the roles of 'consulting physician' and 'responsible physician' in the provision of assisted dying, it frequently mentions the 'health care team' in the context of caring for patients who request euthanasia. Notably, the text explicitly mentions nursing professionals in constant relation to the responsible physician(30). This multi-phase approach was essential to achieve semantic and technical equivalence, as today's recommendation emphasizes the importance of conducting cross-cultural adaptations of measurement tools. This ensures that linguistic aspects, cultural connotations, and variations are adequately addressed, which can significantly affect the feasibility of the tool (Sousa & Rojjanasrirat, 2011; Escobar-Bravo, 2004).*

**7- Reviewer’s comment**: “-Aspects of the pilot test should be explained in more depth (what population was used, characteristics, how these people evaluated the instrument)”.

Authors’ response: While we did not implement a formal pilot test using Likert scale scores or calculate the Content Validity Index (CVI) as outlined, we prioritized expert input to assess the relevance of each item. Given that we were translating an existing instrument rather than creating a new one, we initially assumed content validity based on the established framework of the original scale. Their feedback helped us refine the instrument, ensuring that it accurately captures the attitudes of healthcare professionals. We recognize that we primarily conducted a face validity assessment through expert review. Moving forward, we acknowledge the importance of both content and face validity techniques and will consider incorporating more rigorous validation methods in future studies to enhance the evaluation process of our measurement instrument.

Face validity was established through the assessment of 10 experts with experience and knowledge of the law and its applications in the context of euthanasia in the Balearic community. Following this, a pilot study of the final draft of ATE version 2 was conducted to evaluate the comprehension and suitability of the questionnaire, as well as its cultural appropriateness for the Spanish population. During this phase, the focus was on identifying unclear items, potential errors, and any misinterpretations, while also assessing the effort required to complete the questionnaire. The process was finalized when the professionals reported no significant difficulties, resulting in the final version of the ATE-ES-R questionnaire

**8- Reviewer’s comment**: “The measurements should better explain how each variable was measured, as well as what questions were asked of the participants regarding their knowledge and ethics of euthanasia”.

Authors’ response: Thank you for your constructive feedback regarding the measurement of variables in our study. We appreciate your suggestion to provide more clarity on how each variable was assessed. We have revised the section to better explain how each variable was measured, incorporating your suggestions.

In our manuscript, we included a section on demographic information where participants self-reported their sociodemographic characteristics, such as age, gender, marital status, educational level, nuclear family status, and years of professional experience.Additionally, we incorporated specific questions aimed at evaluating their knowledge of ethics and euthanasia. See also Table 1.

Moreover, in the data analysis section, we included a detailed description of how each variable was handled. A descriptive analysis of the variables was performed to characterize the sample composition, utilizing frequency distributions with percentages for categorical variables, as well as means and standard deviations for quantitative variables.

Demographic information: Participants provided self-reported sociodemographic characteristics, including age, gender, marital status, educational level, nuclear family status, and years of professional experience. Additionally, we included specific questions related to their knowledge of ethics and euthanasia. These questions aimed to assess their understanding of relevant laws and ethical dilemmas encountered in practice. These questions included (Do you know the national law regarding assistance in dying?, Have you attended any training on ethics?, Have you attended any training on euthanasia?, Do you consider yourself adequately trained on euthanasia?) among others

**Data Analysis**

For the statistical analyses, IBM SPSS AMOS version 26, Jamovi version 2.3, and jMetrik version 4.1.1 were utilized. Initially, data coding and exploration were conducted to prepare the dataset for analysis. Subsequently, a descriptive analysis of the variables was performed to characterize the sample composition. Descriptive statistics included frequency distributions with percentages for categorical variables, as well as means and standard deviations for quantitative variables. To assess the normality of quantitative variables, the Kolmogorov-Smirnov test (N>50) was utilized.

**9- Reviewer’s comment**: “When you describe the instrument you refer to the original four-dimensional structure, but you do not refer to whether this structure is from the original version or the Spanish version of Fernandez et al. This is very much related to the factor analysis”.

Authors’ response: Thank you for your valuable feedback regarding the description of the instrument. We would like to clarify that we divided our sample into two groups and conducted a new Exploratory Factor Analysis (EFA) to assess the factor structure of the Spanish version of the Attitude Toward Euthanasia scale.

This analysis allowed us to evaluate the dimensionality of the instrument in our context, ensuring that it aligns with the current attitudes of healthcare professionals. We appreciate your observation, which has prompted us to provide more clarity on our factor analysis results in the manuscript

**10- Reviewer’s comment**: “-I have some important questions on the statistical analysis. To begin with, you do a CFA based on several models. It is essential to explain and reference on the basis of which literature, previous studies or assumptions you test these models, as Fernandez et al. did. Usually the CFA is done on the basis of an EFA, which is then tested on a different sample than the one on which the EFA was performed. If a CFA is done on the basis of an established model(s), this needs to be explained”.

Authors’ response: Thank you for your valuable feedback regarding the statistical analysis. In our study, we opted to divide the sample for the Confirmatory Factor Analysis (CFA), in contrast to the approach taken by Fernández et al., where the CFA was conducted using the same sample. This decision was made to enhance the robustness of our findings and to validate the factor structure across different subsets of our sample. We believe that this approach provides a more rigorous validation compared to using a single sample for both Exploratory Factor Analysis (EFA) and CFA.

**11- Reviewer’s comment**: “-Secondly, the sample adequacy of the factor analysis should be reported (e.g. by reporting the KMO and Barlett's statistic). The type of matrix used (polychoric or pearson) should be reported, determining the approach to the factor analysis that has been carried out (linear or non-linear). This decision is usually made by assessing, among other things, the symmetry and kurtosis of the items (mardia test). The estimation procedure of the model solution (e.g. Unweigthed Least Squares, ULS) or the type of rotation is not shown either. All these aspects should appear in the method. I personally cannot conceive of presenting values without confidence intervals, but I leave this to the editor and other reviewers to assess”.

Authors’ response: Thank you for your insightful feedback regarding the factor analysis methodology. We appreciate the importance of clearly reporting sample adequacy indicators, such as the KMO and Bartlett's test statistics, as well as specifying the type of correlation matrix used (polychoric or Pearson). In our study, we conducted an analysis of the symmetry and kurtosis of the items, including the Mardia test, to determine the appropriate approach for factor analysis. We will ensure that the estimation procedure and type of rotation applied (e.g., Unweighted Least Squares, ULS and Varimax rotation) are clearly detailed in the methods section. Following this suggestion, we have now included the following explanatory text:

An Exploratory Factor Analysis (EFA) was conducted, utilizing the Kaiser-Meyer-Olkin (KMO) measure and Bartlett’s test of sphericity to assess sample adequacy. The Pearson correlation matrix was employed, followed by principal component extraction and Varimax rotation with Kaiser normalization. Reliability was evaluated using Cronbach's α and Omega. After conducting the Exploratory Factor Analysis (EFA), we proceeded with a Confirmatory Factor Analysis (CFA) utilizing the maximum likelihood estimation method. The adequacy of the factorial solution was assessed through several fit indices, including chi-square (X²), Root Mean Square of Residuals (RMSR), Root Mean Square Error of Approximation (RMSEA), Non-Normed Fit Index (NNFI), Comparative Fit Index (CFI), Goodness-of-Fit Index (GFI), and Adjusted Goodness-of-Fit Index (AGFI). An acceptable fit was indicated by an RMSR value of 0.05 or lower. For RMSEA, a value below 0.05 was deemed indicative of a good fit, while values between 0.05 and 0.08 were classified as reasonable. Furthermore, NNFI and CFI values reaching 0.95 or above, along with GFI and AGFI values greater than 0.90, signified a well-fitting model. The AGFI should also surpass 0.90 to suggest optimal model adequacy. All fit indices were expected to fall within the range of 0 to 1, with a benchmark of 0.90. In addition, lower values for the standardized root mean square residual (RMR) and RMSEA were associated with a better fit, with a reference point set at 0.08.Additionally, a parallel analysis was executed to further validate the factor structure. The determination of the number of factors to retain was conducted using parallel analysis, and the reliability of the selected factors was assessed; confidence intervals at 95% were computed for both the item scores and the model metrics.

**12- Reviewer’s comment**: “The reliability analysis should be significantly improved. To begin with, you should provide the omega coefficient, which has many advantages over the Crombach coefficient (both are usually provided). In addition, given that there are models with several dimensions, the reliability values for each dimension should be reported for each model (there are authors who argue that it makes no sense to report a total coefficient in these cases). I am flexible and I do not mind if a total coefficient is reported if there are dimensions in the instrument, but the coefficients of each dimension should always be reported”.

Authors’ response: Thank you for your valuable feedback regarding the reliability analysis. We have included both the omega coefficient and Cronbach's alpha in the revised analysis, as they provide complementary insights into the reliability of the scales. Additionally, I have reported the reliability values for each dimension across the various models, as per your suggestion. This ensures a comprehensive understanding of the reliability within the instrument.

*The reliability of the measurement instrument was evaluated using both Cronbach's Alpha and McDonald's Omega coefficients for all factors across the various models. ( See table 4) The results indicate that Model 1 (M1) and the two factors from Model 2 (M2) exhibited the highest reliability, with both Cronbach's Alpha and Omega values exceeding the acceptable threshold of 0.70. In contrast, Models 3 (M3) and 4 (M4) demonstrated factors with reliability coefficients significantly below 0.70, suggesting that these models may require further refinement to enhance the consistency of the items*.

***Table 5. Cronbach's Alpha and McDonald's Omega Coefficients for Measurement Models***

|  | M1 | M2 | | M3 | | | M4 | | | |
| --- | --- | --- | --- | --- | --- | --- | --- | --- | --- | --- |
|  |  | F1 | F2 | F1 | F2 | F3 | F1 | F2 | F3 | F4 |
| α de Cronbach | 0.889 | 0.832 | 0.841 | 0.868 | 0.838 | 0.670 | 0.868 | 0.794 | 0.769 | 0.670 |
| ω de Mc Donald | 0.892 | 0.839 | 0.846 | 0.868 | 0.839 | 0.670 | 0.868 | 0.794 | 0.770 | 0.670 |

**13- Reviewer’s comment**: “-An analysis by known groups could have been carried out. It would improve the manuscript quite a bit, but I understand this is optional”.

Authors’ response: Thank you for your insightful suggestion regarding the analysis by known groups. While we did not include this analysis in the current study, we recognize its potential to enhance the manuscript. Given the extensive data collected of this study, including information from students and other questionnaires, we plan to conduct it in a future article.

**14- Reviewer’s comment**: “In my opinion in the methodology it should appear how it has been done and in the results the data obtained. In the results it should therefore appear the linguistic changes made as well as the results of the pilot test”.

Authors’ response: Thank you for your feedback. We would like to inform you that we have included the items with the changes made to the questionnaire in a supplemental document accompanying the article. This addition provides a clear overview of the modifications implemented. We also move the development of the questionnaire to a new section in the results as follow:

***Development and Preliminary Evaluation of the ATE-ES-R Questionnaire***

*Prior to finalizing the ATE-ES-R questionnaire, we engaged in a multi-phase process to ensure its relevance and appropriateness for the Spanish context.* *In collaboration with the Health Care Ethics Committee, we modified the expression “the physician” to “the health care team” in all items, reflecting the collaborative nature of end-of-life care. While Law 3/2021 regulating euthanasia in Spain mentions 'consulting physician' and 'responsible physician,' it also emphasizes the involvement of the 'health care team,' explicitly acknowledging nursing professionals alongside physicians(30). To establish preliminary face validity, we consulted 10 experts knowledgeable about the law and its applications in the context of euthanasia in the Balearic community. Following this consultation, we conducted a pilot study of the final draft of the ATE-ES-R questionnaire to evaluate its comprehension, suitability, and cultural appropriateness. During this phase, we identified unclear items and potential misinterpretations, ensuring the questionnaire was user-friendly. The feedback from professionals indicated no significant difficulties, leading to the final version of the ATE-ES-R questionnaire. This multi-phase approach was essential to achieve semantic and technical equivalence, as today's recommendation emphasizes the importance of conducting cross-cultural adaptations of measurement tools. This ensures that linguistic aspects, cultural connotations, and variations are adequately addressed, which can significantly affect the feasibility of the tool*

**15.- Reviewer’s comment**: “The response rate and the number of non-responders should be presented (I agree that the reasons cannot be given for ethical reasons). It is important to know what is the percentage of non-respondents, as non-probability convenience sampling has been carried out and this may affect the accuracy of the results (it should appear in the discussion as a limitation) “.

Authors’ response: We have included the response rate of 16.59% in the manuscript, along with a discussion of the non-responder percentage. We recognize that this is a limitation, particularly given the sensitive nature of the topic and the high workload in the healthcare sector. Additionally, the questionnaire was distributed via the internal work email system, which is not commonly used outside the healthcare setting. We have addressed these factors extensively in the limitations section of the discussion.

**16.- Reviewer’s comment**: “In the CFA, they should report KMO and barlett. My proposal is to present a table showing the fit values of the four models, so that they can be compared. Moreover, in some models we do not have information about the fit values in a complete way (example: CFI in model 1, SRMR of the four models, etc.)”.

Authors’ response: We appreciate the reviewer’s suggestion regarding the reporting of KMO and Bartlett’s test in the confirmatory factor analysis (CFA). We have created a table that presents the fit values for all four models, allowing for direct comparison. This table includes all relevant fit statistics, ensuring a comprehensive overview of the model comparisons.

***Table 4. Fit Indices for CFA Models***

|  | M1 | M2 | M3 | M4 |
| --- | --- | --- | --- | --- |
| Chi-square (χ²) | 124.159 | 85.080 | 67.602 | 109.415 |
| Degrees of freedom (DF) | 30 | 30 | 27 | 29 |
| p-valor | 0.000 | 0.000 | 0.000 | 0.000 |
| CMIMN/DF | 4.139 | 2.836 | 2.504 | 3.773 |
| RMR | 0.052 | 0.040 | 0.034 | 0.039 |
| GFI | 0.941 | 0.960 | 0.968 | 0.964 |
| AGFI | 0.892 | 0.927 | 0.934 | 0.898 |
| CFI | 0.950 | 0.971 | 0.979 | 0.957 |
| IFI | 0.951 | 0.971 | 0.979 | 0.958 |
| NFI | 0.925 | 0.956 | 0.965 | 0.934 |
| TLI | 0.925 | 0.956 | 0.964 | 0.934 |
| RMSEA | 0.090 | 0.069 | 0.062 | 0.085 |
| PCLOSE | 0.000 | 0.035 | 0.129 | 0.000 |
| AIC | 174.159 | 135.080 | 123.602 | 161.415 |
| ECVI | 0.449 | 0.348 | 0.319 | 0.416 |

**17.- Reviewer’s comment**: “Present the above-mentioned regarding the reliability-internal consistency values”.

Authors’ response: We have now added a table with both values.

**18.- Reviewer’s comment**: “The presentation of the tables should be improved, where values with decimals appear with commas and should be replaced by dots. In addition, the clarity of the graphs should be improved”.

Authors’ response: Thank you for your helpful comments regarding the presentation of the tables and graphs. We appreciate your attention to detail. In the revised manuscript, we will ensure that all decimal values are consistently formatted using dots instead of commas, as per the standard conventions in scientific writing.

**19.- Reviewer’s comment**:” They present a validation where few psychometric properties are evaluated (construct validity and reliability -internal consistency). This is a very important limitation that should at least be admitted in the discussion”.

Authors’ response: We have incorporated psychometric properties into the study of this test. However, certain additional analyses could not be conducted as the study had already been completed. These aspects have been addressed in the limitations section of the article."

**20.- Reviewer’s comment**: “This manuscript requires important changes, but they can be implemented. I encourage the authors to do so. Best regards.”

Authors’ response: Thank you for your encouraging feedback regarding our manuscript. We appreciate your constructive comments and acknowledge the importance of the suggested changes. We have already made the necessary revisions, which are attached here for your review. Your support reinforces our commitment to improving the manuscript, and we hope the revised version effectively addresses your concerns.

**Response to Reviewer #2:**

We thank the reviewer for his vigorous appraisal of our work. It what follows we address his comments, point by point.

**1.- Reviewer’s comment**: “The background section adequately establishes the importance and relevance of the topic (Euthanasia). However, the study's specific relevance is not sufficiently explained. It is unclear why a re-evaluation of the Attitude Toward Euthanasia (ATE) scale, previously validated by Fernandez et al, is necessary. The manuscript should highlight any potential shortcomings of the original version and clearly state what new contributions this re-evaluation provides, especially considering that the psychometric properties evaluated in the first version are not poor”.

Authors’ response: The primary reasons for this reevaluation are as follows: First, the introduction of the Euthanasia Law in Spain has significantly altered the social and healthcare context in which attitudes toward euthanasia are formed. Given these changes, it is crucial to reassess the ATE scale to ensure that it remains relevant and accurately reflects current attitudes and perceptions.

Second, while the psychometric properties of the initial version of the ATE scale were acceptable, there were identified areas for improvement. For example, certain items may not adequately capture the nuances of contemporary attitudes shaped by recent legal and societal developments.

We have clarified these points in the revised text and ensured that the objectives stated at the beginning of the introduction align with those presented in the discussion.

We appreciate your constructive feedback, which has allowed us to strengthen the rationale for our study.

The introduction of the Euthanasia Law in Spain has significantly altered the social and healthcare context in which attitudes toward euthanasia are formed. Given these changes, it is crucial to reassess the Attitude Toward Euthanasia (ATE) scale to ensure that it remains relevant and accurately reflects current attitudes and perceptions among healthcare professionals. While the psychometric properties of the initial version of the ATE scale were acceptable, areas for improvement were identified. For instance, certain items may not adequately capture the nuances of contemporary attitudes shaped by recent legal and societal developments. This study aims to address these gaps by validating and adapting the ATE scale for a diverse sample of healthcare professionals in SpainTherefore, the aim of this study has been to revise the first translation/validation of the Attitude Toward Euthanasia scale (ATE) by Fernandez et al. (29) using a larger and more heterogeneous sample of healthcare professionals. This approach will allow for a comprehensive understanding of attitudes across different roles within the healthcare sector. The study aims to identify the different dimensions of the questionnaire and to partially modify certain items to better align with the current cultural context in Spain, particularly in light of the regulation of euthanasia in 2021.Ultimately, the goal is to develop an instrument that will be assessed for its reliability and validity after the study is completed, reflecting the evolving attitudes of healthcare professionals toward euthanasia amidst recent legal changes

**2.- Reviewer’s comment**: “ Additionally, in the sentence, “The number of patients who request or receive MAID is gradually increasing in Spain, since the law was developed a few years ago,” the acronym MAID should be defined, and proper references to the relevant law should be included”.

Authors’ response: We appreciate the reviewer’s comments. We have made the suggested revisions to the text. In the introduction, we have clearly defined the meaning of the acronym MAID (Medical Assistance in Dying) to avoid any ambiguity. Additionally, we have included specific and up-to-date references to Organic Law 3/2021 regulating euthanasia in Spain, citing relevant literature to support our statement about the gradual increase in patients requesting or receiving MAID since the implementation of this law

“There is an ongoing debate on the decriminalization of euthanasia in Spain, which tends to resurface following high-profile media cases. Medical Assistance in Dying (MAID) encompasses both the euthanasia process and assisted suicide, serving as the focal point in the current bioethical debate on end-of-life ethical dilemmas. Since the approval of Spain's Organic Law 3/2021 regulating euthanasia, the number of patients requesting or receiving MAID has been steadily increasing.(20–29). Although this law was developed a few years ago, its implications continue to shape discussions in both medical and ethical fields (30).”

**3.- Reviewer’s comment**: “ Finally, Furthermore, there is an inconsistency between the objective stated at the end of the introduction and the one that appears at the beginning of the discussion “The main objective of the present study was to validate the questionnaire in Spanish after modifying all the items, changing the expression “the physician” in each item to “the health care team” .

Authors’ response: Thank you for your feedback. We have addressed the inconsistency by ensuring that the objective stated at the end of the introduction aligns with the one in the discussion. Both sections now clearly state that the main aim of the study was to validate the questionnaire in Spanish after modifying the items, particularly changing 'the physician' to 'the health care team.' This adjustment was made in light of the recent implementation of the euthanasia law in Spain, reflecting a shift in the sociocultural context surrounding this issue.

**4.- Reviewer’s comment**: “ And, you have stated at the objectives: “Therefore, the aim of this study has been to revise the first translation/validation of the Attitude Toward Euthanasia scale (ATE) by Fernandez et al. (29) with a larger sample of participants, to identify the different dimensions of the questionnaire, to partially modify some of the items to adapt them to the current context of our culture and finally to present an instrument with a good reliability and validity”. These objectives need more accurate elaboration regarding: in what population to be applied: nurses, all health care professionals…? And, at the end of the sentence “finally to present an instrument with a good reliability and validity” there is a “value judgment” as you don’t know if this is like that before the study. So, it must be re-phrased”.

Authors’ response: We have revised the objectives for clarity and specificity. Additionally, we have rephrased the final part of the sentence to avoid any presumption of reliability and validity prior to the study.

**5.- Reviewer’s comment**: “Study Population/sample: The study population and sample (and sample size calculation) must be separated and should be better defined”.

Authors’ response: We have revised the section on the study population and sample to ensure clarity and better definition. The study population now explicitly refers to health care professionals in active within the Balearic Health Service and we have included the response rate of 16.56%.

*Sample*

*All health professionals of the Balearic Health Service and specialist professionals in training in the Balearic Islands were invited to participate in the study. Convenience non-probabilistic sampling was used. The study did not consider probabilistic sampling as its primary objective was to achieve a large sample size. For the recruitment of the sample, once authorized by the different managements, through the respective research commissions, collaboration was requested for the dissemination of the questionnaire by global mailing to all the professionals of the different health services. The response rate for the study was 16.56%*

**6.- Reviewer’s comment**: “Population is supposed to include all health professionals at Balearic health service but must to define what type of health professionals (and also, state that health professionals and health workers comprise the same population). The results section should include the total percentages of the different professions involved, as there may be significant differences between them. The inclusion of Nursing students must be clarified. Why not other health students but yes to a resident (specialist professionals in training programs)? Were all resident (from different professions included)?”.

Authors’ response: Thank you for your valuable feedback. We have clarified that the study population encompasses all health professionals within the Balearic Health Service, specifically detailing the types of professionals included, such as nurses, doctors, and allied health personnel. We ultimately decided to exclude nursing students from the study to focus solely on active professionals. As for the included residents from various specialties, such as MIR (Medical Intern Residents), EIR (Nursing Intern Residents), and PIR (Psychology Intern Residents), all were invited to participate via email. They are considered active professionals despite still completing their specific training in the hospital, ensuring a comprehensive representation of healthcare professionals in our study. We have added this information to he table 1.

**7.- Reviewer’s comment**: “Sample Size Justification: The estimated sample size should be justified with references. Although the sample size appears sufficient for a Confirmatory Factor Analysis (CFA), the rationale that it is 10 subjects per item does not align with the estimated size. The sample size for factor analysis is a highly debated issue, and the outdated concept of 10 subjects per item should be reconsidered. Modern approaches consider the number of items, response ranges, and other factors”.

Authors’ response: Thank you for your insightful comment regarding the sample size estimation for the confirmatory factor analysis (CFA). We acknowledge that the rule of thumb of 10 subjects per item has been widely debated in the literature, and indeed, it is considered outdated by many scholars in psychometrics and factor analysis. Recent studies suggest that sample size requirements should be guided by a combination of factors, including the number of items, the number of response categories, the expected factor loadings, and the complexity of the model (MacCallum et al., 1999; Kline, 2015; Wolf et al., 2013).

In light of these recommendations, we carefully evaluated the sample size in relation to these elements to ensure that it is sufficient to provide reliable and robust CFA results. Our approach aligns with current best practices in the field, which emphasize the importance of a tailored evaluation of sample adequacy rather than relying on rigid rules like the 10-to-1 ratio."

1. Kline, R. B. (2015). *Principles and Practice of Structural Equation Modeling* (4th ed.). The Guilford Press.
2. Wolf, E. J., Harrington, K. M., Clark, S. L., & Miller, M. W. (2013). Sample size requirements for structural equation models: An evaluation of power, bias, and solution propriety. *Educational and Psychological Measurement, 73*(6), 913-934.
3. Ferrando, P.J.; Lorenzo-Seva, U.; Hernández-Dorado, A.; Muñiz, J. Decalogue for the Factor Analysis of Test Items. Psicothema 2022, 34, 7–17.

**8.- Reviewer’s comment**: “Additionally, some of the data provided in that section are results and could be placed at the results section”.

Authors’ response: we have included the items with the changes made to the questionnaire in a supplemental document accompanying the article. This addition provides a clear overview of the modifications implemented. We also move the development of the questionnaire to a new section in the results.

***Development and Preliminary Evaluation of the ATE-ES-R Questionnaire***

*Prior to finalizing the ATE-ES-R questionnaire, we engaged in a multi-phase process to ensure its relevance and appropriateness for the Spanish context.* *In collaboration with the Health Care Ethics Committee, we modified the expression “the physician” to “the health care team” in all items, reflecting the collaborative nature of end-of-life care. While Law 3/2021 regulating euthanasia in Spain mentions 'consulting physician' and 'responsible physician,' it also emphasizes the involvement of the 'health care team,' explicitly acknowledging nursing professionals alongside physicians(30). To establish preliminary face validity, we consulted 10 experts knowledgeable about the law and its applications in the context of euthanasia in the Balearic community. Following this consultation, we conducted a pilot study of the final draft of the ATE-ES-R questionnaire to evaluate its comprehension, suitability, and cultural appropriateness. During this phase, we identified unclear items and potential misinterpretations, ensuring the questionnaire was user-friendly. The feedback from professionals indicated no significant difficulties, leading to the final version of the ATE-ES-R questionnaire. This multi-phase approach was essential to achieve semantic and technical equivalence, as today's recommendation emphasizes the importance of conducting cross-cultural adaptations of measurement tools. This ensures that linguistic aspects, cultural connotations, and variations are adequately addressed, which can significantly affect the feasibility of the tool*

**9.- Reviewer’s comment**: “Inclusion/exclusion criteria: You have mentioned “The inclusion criteria were: to be an active health professional in any of the Balearic Islands health system management offices, to be a nursing degree student or a health professional in specialist training in the Balearic Islands, to understand the language and concepts used in the instrument, and to agree to participate in the study by completing the informed consent form authorizing the use of the information for scientific purposes. The exclusion criteria included health workers who declined participation”. From my point of view are not correct. How do you Check if a participant understands the language and concepts of the instrument, if the questionnaire was sent by e-mail? (only after reading the text, you can realize that this is checked by a number of questions included on sociodemographic section of the questionnaire). This point needs clarification. In addition, inclusion and exclusion criteria are characteristics who have the participants and allow them to participate or not. Agreeing or declining participation are not characteristics, are decisions and a part of recruiting”.

Authors’ response: Thank you for your insightful comments regarding the inclusion and exclusion criteria. I appreciate your concern about assessing participants' understanding of the language and concepts in the questionnaire, particularly since it was distributed via email. While we aimed to gauge comprehension through the sociodemographic questions, I recognize that this approach has limitations in a remote setting. Participants were encouraged to ask for clarification if needed, but I understand that this may not fully address the concern.

Thank you for bringing this to my attention, we have now eliminated this from the manuscript.

*The inclusion criteria were: to be an active health professional in any of the Balearic Islands health system management offices and to agree to participate in the study by completing the informed consent form authorizing the use of the information for scientific purposes. Ethical considerations prevented us from asking for the reasons to decline the participation in the study*

**10.- Reviewer’s comment**: “- Control Mechanisms for Internal Validity: The manuscript should describe the control mechanisms implemented to ensure the internal validity of the results, particularly given that this was a mailing survey. Information on how the survey invitations were distributed (e.g., to which email addresses) and the response rates among different recruitment methods should be included. How were the inclusion criteria addressed and evaluated?”.

Authors’ response: The survey invitations were distributed to specific email addresses associated with health professionals in the Balearic Islands. This targeted approach aimed to effectively reach our intended population. As mentioned previously, we have included the response rates among different recruitment methods in the manuscript to provide insight into the effectiveness of our outreach efforts.

**11.- 1Reviewer’s comment**: “From my point of view the design is incomplete. Is not the usual manner of present the type of research design. Was a cross-sectional survey with only one collection point? Was the design performed including different phases, as usual in this type of studies for validation? For instance: a translation-back translation stage, pilot phase, psychometric validation, etc…”

Authors’ response: Thank you for your feedback regarding the research design. I appreciate your insights and would like to clarify a few points: The study is indeed a cross-sectional survey with a single data collection point. While this approach is common for our research objectives, I understand it may differ from more extensive validation processes typical in some studies.

We included several phases, such as the translation-back translation stage of the questionnaire and an evaluation by professionals regarding the ethical considerations and clarity of the items.

While we did not conduct a test-retest or formal content validity assessment with experts due to time constraints, we prioritized the timely collection of data to avoid delaying insights on a topic that is both novel and minimally applied in other communities. This urgency allows us to provide valuable perspectives that could inform practices across the country as new legislation is being implemented.

Finally, we conducted a thorough analysis of the data, including psychometric evaluations of the questionnaire to ensure its reliability and validity.

**12.- Reviewer’s comment**: “
- Translation Process: I understand that you had conducted a new translation and cultural adaptation of the original English instrument, but a Spanish version exist (Fernandez et al. version). So, we need more information about it, why and how was performed, more than your explanation. Also, details on the number of translators involved in each phase of translation and back-translation should be provided”.

Authors’ response: Thank you for your insightful comment. We would like to clarify that our study was based on the original English version of the Attitude Toward Euthanasia (ATE). Given the recent introduction of the Euthanasia Law in Spain, we considered it was very important to revisit and adapt certain concepts in the questionnaire to ensure their relevance in the current context. We conducted a new translation to enhance cultural and contextual applicability. We have now explained this in text with more details.

*Design*

Based on the ATE scale (39,40), we conducted a new translation to enhance cultural and contextual applicability. The translation process involved two bilingual translators whose native language is Spanish. After the initial translation, a back-translation was performed by a professional translator whose native language is English. Subsequently, the research team reviewed the translated version and a second team, formed by members of the Health Care Ethics Committee of the Ibiza and Formentera Health Area, revised it to obtain a semantic and technical equivalence in appropriate bioethical language.

**13.- Reviewer’s comment**: “Pilot Test Details: The manuscript should elaborate on the pilot test, including the population used, their characteristics, and how these individuals evaluated the instrument. Again, write methods on the section methods and results on results section, don’t mix it”.

Authors’ response: While we did not implement a formal pilot test using Likert scale scores or calculate the Content Validity Index (CVI) as outlined, we prioritized expert input to assess the relevance of each item. Given that we were translating an existing instrument rather than creating a new one, we initially assumed content validity based on the established framework of the original scale. Their feedback helped us refine the instrument, ensuring that it accurately captures the attitudes of healthcare professionals. We recognize that we primarily conducted a face validity assessment through expert review. Moving forward, we acknowledge the importance of both content and face validity techniques and will consider incorporating more rigorous validation methods in future studies to enhance the evaluation process of our measurement instrument.

***Development and Preliminary Evaluation of the ATE-ES-R Questionnaire***

*Prior to finalizing the ATE-ES-R questionnaire, we engaged in a multi-phase process to ensure its relevance and appropriateness for the Spanish context.* *In collaboration with the Health Care Ethics Committee, we modified the expression “the physician” to “the health care team” in all items, reflecting the collaborative nature of end-of-life care. While Law 3/2021 regulating euthanasia in Spain mentions 'consulting physician' and 'responsible physician,' it also emphasizes the involvement of the 'health care team,' explicitly acknowledging nursing professionals alongside physicians(30). To establish preliminary face validity, we consulted 10 experts knowledgeable about the law and its applications in the context of euthanasia in the Balearic community. Following this consultation, we conducted a pilot study of the final draft of the ATE-ES-R questionnaire to evaluate its comprehension, suitability, and cultural appropriateness. During this phase, we identified unclear items and potential misinterpretations, ensuring the questionnaire was user-friendly. The feedback from professionals indicated no significant difficulties, leading to the final version of the ATE-ES-R questionnaire. This multi-phase approach was essential to achieve semantic and technical equivalence, as today's recommendation emphasizes the importance of conducting cross-cultural adaptations of measurement tools. This ensures that linguistic aspects, cultural connotations, and variations are adequately addressed, which can significantly affect the feasibility of the tool*

**14.- Reviewer’s comment**: “Measurement Explanation: The manuscript should better explain how each variable was measured, including the specific questions asked about participants’ knowledge and ethics regarding euthanasia”.

Authors’ response: Thank you for your constructive feedback regarding the measurement of variables in our study. We appreciate your suggestion to provide more clarity on how each variable was assessed. We have revised the section to better explain how each variable was measured, incorporating your suggestions.

In our manuscript, we included a section on demographic information where participants self-reported their sociodemographic characteristics, such as age, gender, marital status, educational level, nuclear family status, and years of professional experience. Additionally, we incorporated specific questions aimed at evaluating their knowledge of ethics and euthanasia. See also Table 1.

Moreover, in the data analysis section, we included a detailed description of how each variable was handled. A descriptive analysis of the variables was performed to characterize the sample composition, utilizing frequency distributions with percentages for categorical variables, as well as means and standard deviations for quantitative variables.

Demographic information: Participants provided self-reported sociodemographic characteristics, including age, gender, marital status, educational level, nuclear family status, and years of professional experience. Additionally, we included specific questions related to their knowledge of ethics and euthanasia. These questions aimed to assess their understanding of relevant laws and ethical dilemmas encountered in practice. These questions included (Do you know the national law regarding assistance in dying?, Have you attended any training on ethics?, Have you attended any training on euthanasia?, Do you consider yourself adequately trained on euthanasia?) among others

**Data Analysis**

For the statistical analyses, IBM SPSS AMOS version 26, Jamovi version 2.3, and jMetrik version 4.1.1 were utilized. Initially, data coding and exploration were conducted to prepare the dataset for analysis. Subsequently, a descriptive analysis of the variables was performed to characterize the sample composition. Descriptive statistics included frequency distributions with percentages for categorical variables, as well as means and standard deviations for quantitative variables. To assess the normality of quantitative variables, the Kolmogorov-Smirnov test (N>50) was utilized.

**15.- Reviewer’s comment**: “Instrument Structure: When describing the instrument, you refer to a four-dimensional structure, That I’m assuming is the English original structure but it is unclear whether this structure pertains to the original or the Spanish version by Fernandez et al. This is closely related to the factor analysis and should be clarified. The CFA is based on several models, but it is essential to explain and reference the literature, previous studies, or assumptions that justify testing these models, like what Fernandez et al. did. Typically, a CFA is conducted following an Exploratory Factor Analysis (EFA), which is then tested on a different sample or if the CFA is based on pre-established models (hypothesis contrasts), this should be clearly explained. If not, it seems that you are exploring different options to see what of them is better fitted, but this is not correct, then, explain why you decided to do a 4 CFA based on different dimensions?”.

Authors’ response: Authors’ response: Thank you for your valuable feedback regarding the statistical analysis. We would like to clarify that, in our study, we opted to divide the sample to perform, first an EFA and with the other half the CFA, in contrast to the approach taken by Fernández et al., where the CFA was conducted using the same sample. This decision was made to enhance the robustness of our findings and to validate the factor structure across different subsets of our sample. We believe that this approach provides a more rigorous validation compared to using a single sample for both Exploratory Factor Analysis (EFA) and CFA.

**17.- Reviewer’s comment**: “Report the sample adequacy measures for factor analysis, such as the Kaiser-Meyer-Olkin (KMO) statistic and Bartlett's test of sphericity. Additionally, indicate the type of correlation matrix used (e.g., polychoric or Pearson) and specify the approach to factor analysis (linear or non-linear), which is often determined by assessing item symmetry and kurtosis (e.g., Mardia's test). The estimation procedure of the model solution (e.g., Unweighted Least Squares, ULS) and the type of rotation should also be specified”.

Authors’ response: Thank you for your insightful feedback regarding the factor analysis methodology. We appreciate the importance of clearly reporting sample adequacy indicators, such as the KMO and Bartlett's test statistics, as well as specifying the type of correlation matrix used (polychoric or Pearson). In our study, we conducted an analysis of the symmetry and kurtosis of the items, including the Mardia test, to determine the appropriate approach for factor analysis. We will ensure that the estimation procedure and type of rotation applied (e.g., Unweighted Least Squares, ULS and Varimax rotation) are clearly detailed in the methods section.

An Exploratory Factor Analysis (EFA) was conducted, utilizing the Kaiser-Meyer-Olkin (KMO) measure and Bartlett’s test of sphericity to assess sample adequacy. The Pearson correlation matrix was employed, followed by principal component extraction and Varimax rotation with Kaiser normalization. Reliability was evaluated using Cronbach's α and Omega. After conducting the Exploratory Factor Analysis (EFA), we proceeded with a Confirmatory Factor Analysis (CFA) utilizing the maximum likelihood estimation method. The adequacy of the factorial solution was assessed through several fit indices, including chi-square (X²), Root Mean Square of Residuals (RMSR), Root Mean Square Error of Approximation (RMSEA), Non-Normed Fit Index (NNFI), Comparative Fit Index (CFI), Goodness-of-Fit Index (GFI), and Adjusted Goodness-of-Fit Index (AGFI). An acceptable fit was indicated by an RMSR value of 0.05 or lower. For RMSEA, a value below 0.05 was deemed indicative of a good fit, while values between 0.05 and 0.08 were classified as reasonable. Furthermore, NNFI and CFI values reaching 0.95 or above, along with GFI and AGFI values greater than 0.90, signified a well-fitting model. The AGFI should also surpass 0.90 to suggest optimal model adequacy. All fit indices were expected to fall within the range of 0 to 1, with a benchmark of 0.90. In addition, lower values for the standardized root mean square residual (RMR) and RMSEA were associated with a better fit, with a reference point set at 0.08.Additionally, a parallel analysis was executed to further validate the factor structure. The determination of the number of factors to retain was conducted using parallel analysis, and the reliability of the selected factors was assessed; confidence intervals at 95% were computed for both the item scores and the model metrics.

**18.- Reviewer’s comment**: “- The reliability analysis requires improvement. The manuscript should include the omega coefficient, which is often preferred over Cronbach’s alpha. For instruments with multiple dimensions, reliability values for each dimension should be reported for each model. While some argue that reporting a total coefficient is unnecessary when dimensions exist, I am flexible on this point, but coefficients for each dimension should always be reported”.

Authors’ response: We have created a table that includes both the omega coefficient and Cronbach’s alpha for each dimension of the various models. This addition provides a more comprehensive view of the reliability of our instruments.

*The reliability of the measurement instrument was evaluated using both Cronbach's Alpha and McDonald's Omega coefficients for all factors across the various models. ( See table 4) The results indicate that Model 1 (M1) and the two factors from Model 2 (M2) exhibited the highest reliability, with both Cronbach's Alpha and Omega values exceeding the acceptable threshold of 0.70. In contrast, Models 3 (M3) and 4 (M4) demonstrated factors with reliability coefficients significantly below 0.70, suggesting that these models may require further refinement to enhance the consistency of the items*.

***Table 5. Cronbach's Alpha and McDonald's Omega Coefficients for Measurement Models***

|  | M1 | M2 | | M3 | | | M4 | | | |
| --- | --- | --- | --- | --- | --- | --- | --- | --- | --- | --- |
|  |  | F1 | F2 | F1 | F2 | F3 | F1 | F2 | F3 | F4 |
| α de Cronbach | 0.889 | 0.832 | 0.841 | 0.868 | 0.838 | 0.670 | 0.868 | 0.794 | 0.769 | 0.670 |
| ω de Mc Donald | 0.892 | 0.839 | 0.846 | 0.868 | 0.839 | 0.670 | 0.868 | 0.794 | 0.770 | 0.670 |

**192.- Reviewer’s comment**: “ Results: As I said, the methodology should detail the procedures, while the results should present the data obtained. Therefore, the results should include the linguistic changes made and the outcomes of the pilot test”

Authors’ response: We appreciate the reviewer’s guidance regarding the separation of methodology and results. In the results section, we have included the linguistic changes made to the questionnaire and the outcomes of the pilot test in a new section called “*Development and Preliminary Evaluation of the ATE-ES-R Questionnaire****”***. This presentation allows for a clearer understanding of the data obtained and its implications for the final version of the ATE-ES-R questionnaire.

**20.- Reviewer’s comment**: “Response Rate: Include the response rate and the number of non-respondents. Even though reasons for non-response cannot be detailed for ethical reasons, the percentage of non-respondents is crucial information. This is particularly important given the non-probability convenience sampling used, which may impact the results’ accuracy and should be discussed as a limitation”

Authors’ response: We have revised the section on the study population and sample to ensure clarity and better definition. The study population now explicitly refers to health care professionals within the Balearic Health Service and we have included the response rate of 16.56%.

*Sample*

*All health professionals of the Balearic Health Service and specialist professionals in training in the Balearic Islands were invited to participate in the study. Convenience non-probabilistic sampling was used. The study did not consider probabilistic sampling as its primary objective was to achieve a large sample size. For the recruitment of the sample, once authorized by the different managements, through the respective research commissions, collaboration was requested for the dissemination of the questionnaire by global mailing to all the professionals of the different health services. The response rate for the study was 16.56%*

**21.- Reviewer’s comment**: “Sociodemographic results: Reading your manuscript is not clear if you are studying all health professionals or only nursing professionals, because too many times refer to nursing specifically. This must be cleared on the text and on the tables-figures. Data on frequencies of different group of professionals is crucial to understand the sample studied and the implications on the results”.

Authors’ response: We appreciate the reviewer’s valuable feedback. We have clarified the study population by providing the specific percentages of the different professions included in our sample. To address the concern regarding variability among professions, we have refined our analysis by dividing the sample into two groups for conducting the EFA and CFA. We have also excluded undergraduate students from the analysis to ensure that all participants are active professionals, representing a wide range of occupations to enrich the study's results. See table 1.

**222.- Reviewer’s comment**: “If “70.2% of the professionals recognize that they have little or no knowledge of the law on the regulation of euthanasia, while 29% have a broad knowledge of it”, then this is contradictory with your inclusion-exclusion criteria that I’ve mentioned before and needs clarification. The same for Health care ethics train”.

Authors’ response: We appreciate the reviewer’s observations regarding the inclusion-exclusion criteria. We have excluded the suggestions related to these criteria, as our aim was to reach all healthcare professionals within the Balearic community. The questions mentioned pertain to another analysis currently being developed using a different questionnaire, which aims to explore the attitudes of healthcare professionals and their potential relationship with knowledge of the euthanasia law and their training in important aspects such as ethics and euthanasia. This distinction clarifies that our current study focuses on the validation and adaptation of the ATE-ES-R questionnaire.

**23.- Reviewer’s comment**: “ CFA Reporting: Include KMO and Bartlett's test results in the CFA, this is important to know about the adequacy of the sample. I recommend presenting a table comparing the fit values of the four models. Currently, some models lack complete information on fit values (e.g., CFI for Model 1, SRMR for all models). In addition, your interpretation and analysis of the data obtained could be subjective and influenced by a tendency to a specific model. For instance: “For these indices, lower values indicate a better fit, with a reference value of 0.08” (referring to a SMRS and RMSA), Why 0.08 and no other? What is the literature who avail that number? The literature says that the closer to Zero the better fit. Nevertheless, data in RMSR is lacking. And, you have to results recommending 1 dimension structure who is in accordance with the theory (first CFA and parallel analysis), then one option is to analyze the data with other perspective, for instance, RASCH analysis and evaluate if the data fits into that model for a 1 dimension”.

Authors’ response: We appreciate the reviewer’s feedback regarding the confirmatory factor analysis (CFA) and the importance of including KMO and Bartlett's test results. In our revised manuscript, we have incorporated these results to assess the adequacy of the sample. Additionally, we have presented a table comparing the fit values for all four models, addressing previous gaps in the data, including CFI for Model 1 and SRMR for all models.

After modifying our sample to exclude students and splitting it for the exploratory factor analysis (EFA) and CFA, our analysis yielded different results. Both Model 1 and Model 2 now show acceptable fit indices, and both the EFA and parallel analysis support Model 2.

Thank you for the suggestion to include Rasch analysis; we have conducted this analysis for both the unidimensional model and for each factor of Model 2, providing further insights into the data's fit

**24.- Reviewer’s comment**: “Reliability Reporting: Present the reliability values as discussed, including values for internal consistency”.

Authors’ response: We have now added a table with both values.

**25.- Reviewer’s comment**: “Tables and Graphs: Improve the presentation of tables, ensuring that values with decimals use periods instead of commas. Additionally, the clarity of graphs should be enhanced”.

Authors’ response: We appreciate the reviewer’s feedback regarding the presentation of tables and graphs. We have revised the tables to ensure that values with decimals now use periods instead of commas. Additionally, we have enhanced the clarity of the graphs to improve their overall presentation.

**26.- Reviewer’s comment**: “ Psychometric Properties: The validation presented evaluates limited psychometric properties (construct validity and internal consistency reliability). This is a significant limitation that should be acknowledged in the discussion. The comments added here could improve this issue and to have a more complete psychometric validation study”.

Authors’ response: We thank the reviewer for highlighting the limitations in our evaluation of psychometric properties. In response to the suggestions from both reviewers, we have conducted additional analyses to enhance the quality of the study. We have improved the evaluation of construct validity and internal consistency reliability. However, we acknowledge that certain aspects of psychometric validation remain unaddressed, and we will clearly outline these limitations in the discussion section of the manuscript

**27.- Reviewer’s comment**: “Discussion and conclusions; These sections are totally influenced by the results, so I don’t have any additional commentary on that, because you must to re-write according with the amendments on the other parts of the manuscript”.

Authors’ response: We have revised and rewritten the Discussion and Conclusion sections according to the new results obtained after the re-evaluation of the questionnaire. These sections now more accurately reflect the current findings and are aligned with the amendments made in other parts of the manuscript.

**28.- Reviewer’s comment**: “Finally, likely you must improve the grammar and the language. For instance, “Mother tongue” could be used but is a “literal” translation, likely “Mother language” or “native language” is better. This is only an example, but there are more sentences that need better redaction”.

Authors’ response: We appreciate the reviewer's attention to the language and grammar of our manuscript. We have carefully reviewed the text and made revisions to enhance clarity and precision. We are committed to improving the overall quality of the writing and have implemented further edits throughout the manuscript to address any remaining issues.

**29.- Reviewer’s comment**: “As my conclusion, this manuscript requires significant revisions, but they are feasible. I encourage the authors to address these suggestions. Re-think on the title of the manuscript, if taken into account my recommendations and something like “Re-evaluation of psychometric properties of … based on…” could be feasible”.

Authors’ response: We thank the reviewer for the valuable feedback and recommendations regarding the title of the manuscript. We agree that a focus on the re-evaluation of the psychometric properties could better reflect the content of our article. Therefore, we propose the revised title: “Re-evaluation of the Psychometric Properties of ATE following Changes in Euthanasia Regulations in Spain"
