# Supplementary material for: Re-evaluation of the psychometric properties of ATE following changes in euthanasia regulations in Spain
Source: PLoS One. 2025 Feb 13;20(2):e0319015. doi: 10.1371/journal.pone.0319015 (PMC11824957; doi:10.1371/journal.pone.0319015)
Supplement: Attachment — Submitted filename: Response to Reviewer.docx [file pone.0319015.s003.docx]

**Response to Reviewer #1:**

Dear Authors

Thank you for your efforts. I believe that the changes implemented have improved the manuscript significantly. You have responded adequately to most of the recommendations and for this I congratulate you. However, given that you have made substantial modifications to the analyses performed, in my opinion there are still some new questions to be answered. Please find attached my recommendations for improvement:

Authors’ response: Thank you very much for your thoughtful review and for your kind words regarding the improvements made to the manuscript. I truly appreciate your acknowledgment of the efforts put into addressing the recommendations, and I am grateful for your constructive feedback.

I am pleased to hear that you believe the changes have significantly enhanced the manuscript. I also recognize the importance of the new questions you’ve raised, particularly in light of the substantial modifications to the analyses. I will carefully review your attached recommendations and work on providing clear and comprehensive answers to ensure that all concerns are thoroughly addressed.

Once again, thank you for your time and valuable input. Your feedback has been essential in guiding the revisions and ensuring the quality of the manuscript.

**1.- Reviewer’s comment**: Modify this expression: The original scale had a Cronbach's Alpha of 0.87.page 25: It is better to express it like this: A Cronbach's Alpha of 0.87 was reported. Indicate in this sentence that the numbers in brackets are the item numbers: example: severe pain [items 1,3,9],

Authors’ response: Done

**2.- Reviewer’s comment**: I think you have confused face validity with content validity. Face validity assesses the comprehensibility, appropriateness and acceptability of the tool from the point of view of the population where it is to be used. The use of experts is generally used to assess content validity. I will be flexible in this respect, as for this instrument the study population and experts are similar, but I note this for future research (ideally you would have used non-expert healthcare for face validity).

Authors’ response: Thank you for your observation; you are absolutely right. I acknowledge the difference between face validity and content validity, and I appreciate your clarification. I will take this into account for future research.

**3.- Reviewer’s comment**: You state in your answer that you have divided the sample to perform the EFA and then the CFA. But the system you used to divide the sample does not appear in the manuscript (you usually use systems that ensure the homogeneity of the two sub-samples: e.g. the SOLOMON system of Lorenzo Seva and Ferrando). The system used for this sample division should appear in the text. But more importantly, the results do not show how many subjects were included in the EFA and how many in the CFA. In fact, the KMO of the sample in the CFA does not appear either. Furthermore, the models tested in the CFA should be justified with references. It is accepted that your EFA indicates two dimensions and therefore this model is tested, but the one-, three- and four-factor models are not justified or referenced.

Authors’ response: Thank you for pointing out the absence of a detailed explanation of the sample division system. We acknowledge the importance of using a method that ensures the homogeneity of the sub-samples, such as the SOLOMON system proposed by Lorenzo-Seva and Ferrando.

We have now clarified this in the manuscript “To conduct the exploratory and confirmatory factor analyses, the sample was divided into two parts using the Solomon method, which ensures that the two subsamples share equivalent levels of common variance. The sample size for EFA was 389 and the sample size for CFA was 389”.

(40) Lorenzo-Seva U, Ferrando PJ. FACTOR: A computer program to fit the exploratory factor analysis model. *Behav Res Methods*. 2022;54(1):472-78.

Also in the CFA section we have added this text:

“In the confirmatory factor analysis performed in this study (n= 389) and in addition to the two-factor model suggested by the EFA, one-, three-, and four-factor models were tested in the CFA. The one-factor model was included as a baseline to examine the plausibility of a single general dimension explaining all items. The three- and four-factor models were tested based on theoretical and empirical considerations from previous research on similar instruments (39,41,60), where multidimensional structures have been observed. Testing these models allowed for a comprehensive comparison to confirm that the two-factor solution provided the best fit”

The Kaiser-Meyer-Olkin (KMO) measure was 0.873, and Bartlett’s test of sphericity was statistically significant (p = 0.000).

**4.- Reviewer’s comment**: In table 4 in the table footer you should put the meaning of each index for a better understanding.

Authors’ response: Thank you for your suggestion. I have added the meaning of each index in the footer of Table 4 to improve clarity and understanding.

*CMIN/DF (Chi-Square/df); RMR (Root Mean Square Residual);GFI (Goodness of Fit Index); AGFI (Adjusted Goodness of Fit Index); NFI (Normed Fit Index); IFI (Incremental Fit Index); TLI (Tucker-Lewis Index); CFI (Comparative Fit Index); RMSEA (Root Mean Square Error of Approximation); AIC (Akaike Information Criterion); ECVI (Expected Cross-Validation Index)*

**5.- Reviewer’s comment** -You have used a RASCH approach, for which I congratulate you, as it is a methodology that few researchers have mastered. However, there are aspects that raise doubts in my mind:
-The RASCH methodology starts from one premise and that is the unidimensionality of the model and the items. Therefore it is justified to perform a rasch for the one-dimensional model, but I do not understand how to perform a rasch for the two-dimensional model, as this goes against a basic assumption of the rasch. There are some recent articles that defend the combination of factor analysis and rasch, but always based on the performance of unidimensionality analysis, which in your case I do not appreciate. This is very important and is not clarified or explained in your manuscript. If you cannot explain it, my opinion is that it is better just to present the rasch for the one-dimensional model. Additionally you should support with references the fit values of the infit-outfit.

Authors’ response: Thank you very much for your insightful comment. I completely agree with you regarding the unidimensionality assumption of the Rasch model. Upon reflecting on your observation, I have decided to apply the Rasch analysis solely to the unidimensional model, as this aligns better with the core principles of the methodology. We have revised the manuscript to clarify this point.

**6.- Reviewer’s comment** -Another basic assumption of the RASCH is the local independence of the items. This is usually checked with Yen's Q3 test, the correlation matrix of the residuals being provided as evidence for this independence. Therefore, please perform this test and indicate the threshold values set for it.
The manuscript has improved, but these issues need to be resolved. Best regards.

Authors’ response: Thank you very much for your insightful comment. I appreciate your attention to detail regarding the Rasch analysis. I understand that to ensure the validity of the Rasch model, it is essential to demonstrate unidimensionality and local independence of items.

As requested, I have performed an analysis of the residual correlations between the items to evaluate local independence using the Q3 Yen index. The results showed that most of the residual correlations between items are below 0.2-0.3, indicating that the assumption of local independence is met.

In the article we have presented a table with the residual correlations between the items of the questionnaire. The low values in most of the correlations suggest that the items are locally independent.

Additionally, I have included a figure of the Wright map and the true score-theta distribution sigmoid curve as requested, which illustrate the fit of the Rasch model to the data and provide a better understanding of item functioning and participant ability distribution.

“To ensure the validity of the Rasch model for the polytomous items in this instrument, we first confirmed unidimensionality through exploratory factor analysis. Additionally, to verify local independence, we calculated Q3 Yen’s indices for the residual correlations between items, ensuring that no correlation exceeded the threshold of 0.2-0.3. A Wright map and the true score-theta distribution sigmoid curve are also provided to illustrate the model’s fit to the data and further validate the assumptions ( See table 7 and figure 4)”

**Response to Reviewer #2:**

Reviewer #2: Dear Authors,

Thank you very much for taking into account all the comments made by this reviewer. The new version of your manuscript partially reflect our comments and answers but some of them remains lacking.
I'm sure that the manuscript will improve and finally will be published but must to be well prepared in that way.
- Thanks for improving the introduction section and objectives ads mentioned.
- My main concern is based on methods and their reflection on results. You have made an effort to fulfill our recommendations but some of them are not reflected on the text, at least as you answered on the responses to reviewers, for instance:

Authors’ response: First of all, I would like to sincerely thank you for taking the time to review my manuscript and for your thoughtful and constructive comments. I have learned a great deal from your feedback, particularly in the areas of validation and methodological clarity. Your suggestions have been invaluable in improving the quality of the manuscript.

I appreciate your positive remarks regarding the improvements made to the introduction and the objectives section. I will continue to refine the text based on your advice.

Regarding your main concern about the methods and their reflection in the results, I fully understand your points. While I have made an effort to address your suggestions, I realize that some aspects may not have been adequately reflected in the revised version. I will revisit these sections carefully to ensure the methods are clearly aligned with the results, and I will make the necessary adjustments to clarify this relationship further.

Once again, thank you for your time and insights. I am confident that with your guidance, the manuscript will be significantly improved and ready for publication.

**1.- Reviewer’s comment**. You have mentioned that the sample was divided into 2 samples, one for the EFA and other for the CFA, but this remain unwritten on the manuscript and is relevant to know by the readers. You must to provide information on how the sample was divided (Salomon's methods,...) and the sample size of each subsample. And also, there are incongruences on those data, as you have mentioned on the parallel analysis that all the sample was used for that (and the parallel analysis is a part of the EFA analysis). In other words, you can use 2 methods for decide the number of factors at the EFA, the Eigenvalues or parallel analysis, but both are based in the same sample, and on the text is not clear.

Authors’ response: Thank you for pointing out the absence of a detailed explanation of the sample division system. We acknowledge the importance of using a method that ensures the homogeneity of the sub-samples, such as the SOLOMON system proposed by Lorenzo-Seva and Ferrando.

We have now clarified this in the manuscript “To conduct the exploratory and confirmatory factor analyses, the sample was divided into two parts using the Solomon method, which ensures that the two subsamples share equivalent levels of common variance. The sample size for EFA was 389 and the sample size for CFA was 389”.

(40) Lorenzo-Seva U, Ferrando PJ. FACTOR: A computer program to fit the exploratory factor analysis model. *Behav Res Methods*. 2022;54(1):472-78.

Additionally we have now moved the parallel analysis to the section of the EFA.

**2.- Reviewer’s comment** 2. Regarding the above mentioned, I feel that the different subsections on the text must to be in other order or combined. As I mentioned, Parallel analysis is a method to decide how many factor retain at EFA, so it must to be at the same section of EFA analysis and mention that you have made both approaches, based on Eigenvalues and parallel analysis to decide how many factors and models you will try to confirm.

Authors’ response: Thank you ver much for pointing this. We have now moved the parallel analysis to the section of the EFA. Thank you very much for pointing this

**3.- Reviewer’s comment**. Yo say that an analysis of asymmetry and kurtosis, also a Mardia's test was performed to decided what type of correlation matrix to use, but there is a lack of data information to know if a Pearson's Matrix fits the data. In that way, we must to assume that the data are correct. Did you test a polycoric matrix?

Authors’ response: Thank you for your valuable comment. In the initial analysis, we used a Pearson correlation matrix because the data met the basic assumptions required for this approach, including normality. We have now included a more detailed description of the assumptions and data characteristics in the manuscript to clarify the reasoning behind the choice of correlation matrix.

“In the exploratory factor analysis (EFA), the Pearson correlation matrix was utilized after assessing the skewness and kurtosis of the items using Mardia's test. This approach was chosen because the data met the basic assumptions required for Pearson correlation, including normality. We opted for consistency with previous studies that employed the same method.

**4.- Reviewer’s comment**. Some of the figures or tables are not where the text appear the title.

Authors’ response: Thank you for pointing this out. I apologize for the oversight. Unfortunately, due to technical limitations, I was unable to upload the figures in the manuscript file itself and had to submit them separately. I have now made sure that the figures and tables are referenced in the correct order within the text, and I kindly ask for your understanding regarding the separate submission of the figures.
**5.- Reviewer’s comment**. Thank for taking into account my comment about other types of analysis like RASCH, but if you provide, please, make it with all the information. Provide information about the Rasch model used for polytomous items like this instrument. And provide data that the RASCH analysis could be performed. You must to assure two premises: unidimensionality and local independence of items. for the second, usually, a Q3 Yen's indices must to be provided. If there is a high correlation between residual correlations (up to 0.2-0.3), RASCH analysis could not be ideal, but we don't have information about those data. Could you provide a figure of Wright graph and a typical true score - theta distribution sigmoid curve?

Authors’ response: Thank you very much for your insightful comment. I appreciate your attention to detail regarding the Rasch analysis. I understand that to ensure the validity of the Rasch model, it is essential to demonstrate unidimensionality and local independence of items.

As requested, I have performed an analysis of the residual correlations between the items to evaluate local independence using the Q3 Yen index. The results showed that most of the residual correlations between items are below 0.2-0.3, indicating that the assumption of local independence is met.

In the article we have presented a table with the residual correlations between the items of the questionnaire. The low values in most of the correlations suggest that the items are locally independent.

Additionally, I have included a figure of the Wright map and the true score-theta distribution sigmoid curve as requested, which illustrate the fit of the Rasch model to the data and provide a better understanding of item functioning and participant ability distribution.

“To ensure the validity of the Rasch model for the polytomous items in this instrument, we first confirmed unidimensionality through exploratory factor analysis. Additionally, to verify local independence, we calculated Q3 Yen’s indices for the residual correlations between items, ensuring that no correlation exceeded the threshold of 0.2-0.3. A Wright map and the true score-theta distribution sigmoid curve are also provided to illustrate the model’s fit to the data and further validate the assumptions ( See table 7 and figure 4)”.

***Table 7.*** ***Residual correlations between the items of the questionnaire.***

ate1	ate2	ate3	ate4	ate5	ate6	ate7	ate8	ate9	ate10

ate1	1.0000	-0.1798	0.1898	-0.1671	-0.2402	-0.2069	-0.1251	-0.0958	-0.1600	0.1191

ate2	-0.1798	1.0000	-0.0306	0.1290	0.0466	-0.2478	-0.0436	-0.0997	-0.2200	-0.1548

ate3	0.1898	-0.0306	1.0000	-0.2378	-0.1161	-0.2286	-0.1482	0.1352	-0.2109	-0.0697

ate4	-0.1671	0.1290	-0.2378	1.0000	0.0701	-0.1673	0.1095	-0.1910	-0.2252	-0.0875

ate5	-0.2402	0.0466	-0.1161	0.0701	1.0000	-0.1483	0.2086	-0.1904	-0.1640	-0.1752

ate6	-0.2069	-0.2478	-0.2286	-0.1673	-0.1483	1.0000	-0.2445	-0.1299	0.1630	-0.1804

ate7	-0.1251	-0.0436	-0.1482	0.1095	0.2086	-0.2445	1.0000	-0.2051	-0.1111	-0.2170

ate8	-0.0958	-0.0997	0.1352	-0.1910	-0.1904	-0.1299	-0.2051	1.0000	-0.2141	0.2910

ate9	-0.1600	-0.2200	-0.2109	-0.2252	-0.1640	0.1630	-0.1111	-0.2141	1.0000	-0.2631

ate10	0.1191	-0.1548	-0.0697	-0.0875	-0.1752	-0.1804	-0.2170	0.2910	-0.2631	1.0000

**Figure 4: Wright graph showing the relationship between theta and true score.**
