# Supplementary material for: Re-evaluation of the psychometric properties of ATE following changes in euthanasia regulations in Spain
Source: PLoS One. 2025 Feb 13;20(2):e0319015. doi: 10.1371/journal.pone.0319015 (PMC11824957; doi:10.1371/journal.pone.0319015)
Supplement: Attachment — Submitted filename: Response to Reviewer (1)_.docx [file pone.0319015.s004.docx]

**Response to Reviewer #1:**

Dear authors:
Thank you for the work done and the changes implemented. I would like to ask you some questions that I have doubts about:

**1.- Reviewer’s comment**: You indicate the use of the SOLOMON system to divide the sample. Please indicate how it was carried out (statistical programme used) and the Ratio Communality Index obtained.

Authors’ response:

Thank you for your comment. The sample was divided using the SOLOMON method with the FACTOR software, Release version 12.04.05 x 64 bits. The Communality Ratio Index obtained was 0.98. The KMO values for each sample were 0.870 for the first sample and 0.853 for the second sample.

**2.- Reviewer’s comment**: Since you used the mardia test to ensure the convenience of using a pearson matrix, provide the values of symmetry and kurtosis of the items.

Authors’ response:

Thank you very much for your valuable suggestion. We conducted the Mardia test to assess the appropriateness of using a Pearson correlation matrix. Based on the test results, both the asymmetry and kurtosis indices for the items were below 1, which indicated that the distributions were sufficiently normal. Therefore, we proceeded with Pearson's correlation as recommended in the literature for cases with acceptable skewness and kurtosis values.

We greatly appreciate your input regarding the use of polychoric correlations for distributions with more pronounced skewness or kurtosis. Rest assured, we will certainly take this into account in future studies if we encounter distributions that meet those criteria.

**3.- Reviewer’s comment**: Indicate that you confirmed the unidimensionality from the EFA, but in this the analysis identified two factors: ‘Regarding the total explained variance, the analysis identified two factors that together account for 64.51% of the total variance of the construct studied. The first factor explains 52.07% of the variance, while the second factor accounts for 12.43%’. There are other types of analyses to assess the unidimensionality of an instrument. Include objective indices that justify the unidimensionality of the model and therefore the convenience of carrying out a Rach analysis.

Authors’ response:

Thank you for your thoughtful feedback and suggestions. In the previous version of the manuscript, we incorporated a Rasch analysis based on the recommendation of the reviewers to evaluate the unidimensionality of the instrument. At that time, the exploratory and confirmatory factor analyses, as well as the Parallel Analysis, supported a unidimensional structure, which justified conducting the Rasch analysis.

To ensure the robustness of the findings, we divided the sample into two groups: one for conducting exploratory factor analysis (EFA) and the other for confirmatory factor analysis (CFA). The results obtained from these analyses no longer supported a unidimensional structure but instead indicated a two-factor model. Parallel Analysis further reinforced the presence of two factors.

Following , and in line with the recommendations from the initial review, we performed a Rasch analysis on the entire scale under the assumption of unidimensionality. Additionally, consulting further literature, we applied separate Rasch analyses to each of the two factors identified in the model. While the item fit within each factor was acceptable, the overall assumptions of unidimensionality for Rasch modeling were not sufficiently met.

Given the current evidence of multidimensionality and the guidance from a reviewer, we decided to exclude the unidimensional Rasch analysis and the subsequent factor-specific Rasch analyses from this revised version. Instead, we have focused on discussing the psychometric properties of the instrument within the framework of a two-factor model, which aligns better with the data.

We hope this explanation clarifies our approach and rationale, and we welcome further feedback to refine the manuscript further.

Final del formulario

**Response to Reviewer #2:**

Dear Authors, I hope that you have enjoyed the Christmas holidays and wish you a Happy 2025. I just read the comments and the new version of your manuscript that, for sure, is getting better.

Thanks for taking in consideration all the comments. My intention is to improve your manuscript and, that, finally will be published.

I have minor comments that must to be addressed to be ok for publication:

- First of all, please, take care when made changes and be confident that all if well approached. For instance, on the revised manuscript you have:

**1.- Reviewer’s comment**:

"Analysis of Rasch Models: Unidimensional and Two-Factor Approaches. We conducted a Rasch analysis to evaluate the fit of items within both a unidimensional
model and a two-factor model".

But this is not according with the comments and the new supposed version, as you said that only will provide Rasch analysis for the 1 factor model to analyze unidimensionality. Please, read carefully your manuscript and re-write according with the comments.

Authors’ response:

Thank you for your observation. As mentioned previously, the sentence referencing a Rasch analysis for both the unidimensional and two-factor models was mistakenly left from an earlier version and does not reflect the current approach.

In this revised version, we have excluded the unidimensional Rasch analysis, as the results no longer support this structure. Following the recommendations of both reviewers, we conducted Rasch analyses for each factor of the two-factor model to explore their properties further. While these analyses provided some useful insights, they ultimately did not meet the global assumptions of unidimensionality required for Rasch modeling.

**2.- Reviewer’s comment**:

- If you said that something is made, please, write what was made at methods section and provide only results at results section. Below, some examples:
- What software was used to implement Solomon method to split the sample in two sub-samples?

Authors’ response: Thank you for your comment. The sample was divided using the SOLOMON method with the FACTOR software, Release version 12.04.05 x 64 bits. The Communality Ratio Index obtained was 0.98. The KMO values for each sample were 0.870 for the first sample and 0.853 for the second sample.

**3.- Reviewer’s comment**: Please state at methods, assuming that the instrument is based in a likert scale, that "RASCH analysis was made under an Andrich's Rating Scale Model for polytomous Items" (reference, for instance: Meyer J.P. Applied Measurement with JMetrik. Routledge: New York, 2014)

**4.- Reviewer’s comment**: Please, include in methods section all the information about how the RASCH analysis was made. You have written on the results but not how was made at methods. Include a sentence about the analysis to verify "local independence of items", about Wright graph and Characteristic curve, etc,... A numbered list of how was made will be excellent.

**5.- Reviewer’s comment**:Looking at the results of the RASCH analysis, this is not the best, based on the metrics provided. Please, as I mentioned before, state at methods section what type of analysis was made: unfit-outfit, difficulty, reliability and separation indexes for both persons and items, Q3 Yen coefficient-index, graphs,... After that present all data on the results section and, finally, discuss the results on Discussion section. I'm realized that nothing is mentioned on discussion about RASCH analysis, indeed, nothing is discussed about the 1 factor model. You have data to mention that "based on the RASCH analysis, the one factor model have a good fit to the model with exception to quality statistics (reliability and separation index) who..."

Authors’ response comments 3,4 and 5:

Thank you for your detailed observations regarding the Rasch analysis and for your valuable suggestions to improve this aspect of the manuscript. In light of the revised findings of our study, we determined that a unidimensional Rasch analysis was no longer appropriate due to the emerging evidence of a two-factor structure. As a result, we have removed the Rasch analysis from the manuscript entirely, as it does not align with the multidimensional nature of the data.

In previous revisions, and following the reviewers' recommendations, we conducted Rasch analyses on both the unidimensional and two-factor models. However, the global assumptions of unidimensionality for Rasch modeling were not sufficiently met, even within each factor of the two-factor model. Consequently, we have opted to focus on the psychometric properties of the instrument within the framework of a two-factor structure, as supported by exploratory and confirmatory factor analyses and Parallel Analysis.

We are especially grateful for your thoughtful suggestions, including the use of the Andrich's Rating Scale Model and the additional analyses you proposed, such as local independence verification, Wright graphs, and the Q3 Yen coefficient. These ideas have been invaluable and have provided us with insights that will undoubtedly guide future work. However, given that the Rasch analysis is no longer part of this revised manuscript, we believe these recommendations fall outside the scope of the current version.

We have revised the manuscript to ensure consistency with this approach, removing any references to the Rasch analysis. The discussion has been updated accordingly to reflect the focus on the two-factor model.

We hope this explanation addresses your concerns, and we remain open to any further suggestions to improve the clarity and quality of our work.
