# Supplementary material for: Re-evaluation of the psychometric properties of ATE following changes in euthanasia regulations in Spain
Source: PLoS One. 2025 Feb 13;20(2):e0319015. doi: 10.1371/journal.pone.0319015 (PMC11824957; doi:10.1371/journal.pone.0319015)
Supplement: Attachment — Submitted filename: Response to Reviewer.docx [file pone.0319015.s005.docx]

**Response to Reviewer #1:**

Reviewer #1: Dear authors:
Thank you for your efforts throughout the review process. I know it has been hard and required a lot of effort on your part, I hope that at least you have found it useful for future research and that you will take my comments as what they are, an opportunity for improvement. After reading the latest version, I consider that the manuscript can be published, since the comments have been answered and this version is more consistent. I am only going to make a couple of stylistic comments that need to be adjusted. On the other hand, I recommend that in future validations you use the FACTOR program for the factor analysis, not only for the sample splitting procedure, which provides advanced factor analysis, providing for example also confidence intervals for the calculated indices or factor loadings, as well as an analysis of the possible unidimensionality of the model, which can help you to approve other approaches (e.g. Rasch analysis).

Authors’ response: Thank you very much for your kind words and for all the work you have done throughout the review process. We sincerely appreciate the time and effort you have dedicated to our manuscript. Your insightful comments and suggestions have been invaluable, not only for improving this article but also for advancing our knowledge of validation processes.

Indeed, the current version of the manuscript is vastly improved compared to what we initially submitted, and this transformation is largely thanks to your thoughtful feedback. Throughout this process, we have learned an extraordinary amount about validation procedures, and this has already begun to influence our current and future research.

As you noted, we are currently validating another instrument, and the lessons we have taken from your suggestions are enabling us to approach it with much greater rigor and precision right from the start. We also want to thank you for recommending the FACTOR program for advanced factor analysis; we are eager to explore its capabilities further and incorporate it into our future validations.

Once again, thank you for helping us grow as researchers. Your constructive feedback has been an invaluable learning experience for us, and we are truly grateful for your contribution.

**1.- Reviewer’s comment**:
-In the sentence ‘Two items were reverse coded to check for response bias’ indicate which are the items with reverse scoring (it would be sufficient to provide the item number).

Authors’ response: Thank you for your observation. The items that were reverse coded to check for response bias are item 6 and item 9. We have now added this information to the manuscript for greater clarity and transparency.

Two items were reverse coded to check for response bias (item 6 and item 9).

**2.- Reviewer’s comment**: -In the Data analysis section, the reference to the jMetrik software version 4.1.1. should be deleted, as the Rasch analysis has finally been eliminated. The FACTOR program, used to carry out the SOLOMON procedure, should be introduced instead (it is more appropriate to mention the FACTOR program in the method than in the results).

Authors’ response: Thank you for your suggestion. As per your recommendation, we have removed the jMetrik software version 4.1.1. in the Data Analysis section and we have introduced the mention of the FACTOR program in the Methods section.

For the statistical analyses, IBM SPSS AMOS version 26, Jamovi version 2.3, and the FACTOR program were utilized

**3.- Reviewer’s comment**: -In table 1 it is better to put the standard deviation in brackets after the mean; M(SD). You should add footnotes explaining these abbreviations.

Authors’ response: Done

42.94 (±10.73)

*M (mean) SD ( standard deviation) F(frequency)*

**Response to Reviewer #2:**

Thank you very much, again, for your efforts in applying the reviewer’s suggestions and to going better in your manuscript. The decision on delete/eliminate of the manuscript some analysis made is debatable but understandable. For instance, the decision of not provide data on the RASCH analysis. But I’m accepting this decision based on your answer.

Authors’ response: Thank you very much for your kind words and for taking the time to review our manuscript so thoroughly. We deeply appreciate your constructive feedback, which has greatly contributed to improving the quality of our work.

This process has been a significant learning experience for us, and it has already influenced the way we approach our current and future research. We are now validating another instrument, and the insights we have gained through your detailed comments are allowing us to approach it with greater precision and care from the outset.

We are truly grateful for the effort and dedication you have shown throughout this process. Thank you again for your thoughtful contributions and for helping us grow as researchers.

**1.- Reviewer’s comment**: Please, re-write the manuscript and put all the information regarding methods on the manuscript methods: all data and information regarding, for instance, the rationale to test different factors models, Mardia’s test, Solomon procedure, parallel analysis, etc,… must to be written at te methods section and in a convenient order related to the procedure. This recommendation is not new and not addressed on the manuscript.

Authors’ response: Thank you for your valuable feedback. As per your suggestion, we have moved all the information regarding the rationale for testing different factor models, Mardia’s test, the Solomon procedure, parallel analysis, and other relevant details to the Methods section. This information is now presented in a logical and sequential order that reflects the procedural flow of the analyses.

We appreciate your careful attention to this matter, and we believe the manuscript is now more organized and clear.

**Data Analysis**

For the statistical analyses, IBM SPSS AMOS version 26, Jamovi version 2.3, and the FACTOR program were utilized. Initially, data coding and exploration were conducted to prepare the dataset for analysis. Subsequently, a descriptive analysis of the variables was performed to characterize the sample composition. Descriptive statistics included frequency distributions with percentages for categorical variables, as well as means and standard deviations for quantitative variables. To assess the normality of quantitative variables, the Kolmogorov-Smirnov test (N>50) was utilized.

Exploratory Factor Analysis

To conduct the exploratory and confirmatory factor analyses, the sample was divided into two parts. To ensure that the two subsamples share equivalent levels of common variance we used the Solomon method with the FACTOR software, Release version 12.04.05 x 64 bits. The Communality Ratio Index obtained was 0.98. The KMO values for each sample were 0.870 for the first sample and 0.853 for the second sample. The sample size for EFA was 389 and the sample size for CFA: 389 (40). An Exploratory Factor Analysis (EFA) was conducted, utilizing the Kaiser-Meyer-Olkin (KMO) measure and Bartlett’s test of sphericity to assess sample adequacy. The Pearson correlation matrix was computed following the assessment of item skewness and kurtosis using Mardia's test (p-value = 0.643) (see Table 2). Subsequently, principal component extraction was performed, followed by Varimax rotation with Kaiser normalization. This approach was selected as the data met the basic assumptions required for Pearson correlation, including normality.

Parallel Analysis

A parallel analysis was executed to further validate the factor structure. The determination of the number of factors to retain was conducted using parallel analysis, and the reliability of the selected factors was assessed; confidence intervals at 95% were computed for both the item scores and the model metrics.

Confirmatory Factor Analysis

We proceeded with a Confirmatory Factor Analysis (CFA) utilizing the maximum likelihood estimation method. The adequacy of the factorial solution was assessed through several fit indices, including chi-square (X²), Root Mean Square of Residuals (RMSR), Root Mean Square Error of Approximation (RMSEA), Non-Normed Fit Index (NNFI), Comparative Fit Index (CFI), Goodness-of-Fit Index (GFI), and Adjusted Goodness-of-Fit Index (AGFI). An acceptable fit was indicated by an RMSR value of 0.05 or lower. For RMSEA, a value below 0.05 was deemed indicative of a good fit, while values between 0.05 and 0.08 were classified as reasonable. Furthermore, NNFI and CFI values reaching 0.95 or above, along with GFI and AGFI values greater than 0.90, signified a well-fitting model. The AGFI should also surpass 0.90 to suggest optimal model adequacy. All fit indices were expected to fall within the range of 0 to 1, with a benchmark of 0.90. In addition, lower values for the standardized root mean square residual (RMR) and RMSEA were associated with a better fit, with a reference point set at 0.08.

Reliability was evaluated using Cronbach's α and Omega.

**2.- Reviewer’s comment**: Please, when use abbreviations, for instance, on the tables, provide complete names as a foot note on the tables. Some of them are in that way and others not. For this reviewer, it’s important that you address our recommendations, not only in your answers but in the manuscript. I hope to receive a final version with those comments to, finally, accept it.

Authors’ response: Thank you for your valuable comments. We have carefully reviewed and revised all the tables in the manuscript. As per your recommendation, we have ensured that all abbreviations are fully spelled out in the footnotes of the tables. We believe these adjustments address your concerns, and we hope the final version now meets the expectations for acceptance. Thank you again for your thorough feedback and support throughout the review process.

Table 1: *M (mean) SD ( standard deviation) F(frequency)*

Table 2: *M(mean) IC 95%(Confidence interval) DT(standard deviation) As(Asymmetry) Kr(kurtosis)*

Table 3:*M1(model1) M2(model2) M3 (model3) M4 (model4) F1(factor 1) F2(factor 2) F3(factor 3) F4(factor 4)*
